# ASPEN: Robust detection of allelic dynamics in single cell RNA-seq

**DOI:** 10.1371/journal.pcbi.1013837

**Published:** 2025-12-19

**Authors:** Veronika Petrova, Muqing Niu, Thomas S. Vierbuchen, Emily S. Wong

**Affiliations:** 1 Division of Molecular, Structural, and Computational Biology, Victor Chang Cardiac Research Institute, Darlinghurst, Australia; 2 School of Biotechnology and Biomolecular Sciences, University of New South Wales, Sydney, Australia; 3 Developmental Biology Program, Sloan Kettering Institute for Cancer Research, New York, New York, United States of America; 4 Center for Stem Cell Biology, Sloan Kettering Institute for Cancer Research, New York, New York, United States of America; ., CANADA

## Abstract

Single-cell RNA-seq data from F1 hybrids provide a unique framework for dissecting complex regulatory mechanisms, but allelic measurements are limited by technical noise due to low counts. Here, we present ASPEN, a statistical method for modeling allelic mean and variance in single-cell transcriptomic data. ASPEN combines a sensitive mapping pipeline  with a moderated beta-binomial model and adaptive shrinkage to distinguish allelic imbalance and changes to allelic variance in single cells. In both simulated and empirical datasets, ASPEN achieves a ~30% increase in sensitivity over existing approaches for single-cell allelic imbalance detection. Applied to mouse brain organoids and T cells, ASPEN identifies genes with incomplete X inactivation, random monoallelic expression, and significant deviations in allelic variance. These results reveal reduced variance in essential genes, consistent with tight regulatory control, and increased variance at neurodevelopmental and immune loci, indicative of regulatory flexibility.

## Author summary

Hybrid organisms inherit two distinct copies (alleles) of each gene, one from each parent. By measuring how much each allele is expressed in individual cells, we can detect regulatory differences that are invisible in bulk tissue samples. However, single-cell measurements are sparse, making it difficult to separate true biological signals from technical noise. We developed ASPEN, a statistical framework that stabilizes these noisy measurements to reliably estimate allelic expression. ASPEN improves the detection of allelic imbalance by up to 30% compared with existing methods and, uniquely, quantifies allelic variance, how much allele usage fluctuates across cells. Applying ASPEN to developing brain cells and immune cells, we discovered that essential “housekeeping” genes maintain remarkably stable allelic ratios, while genes involved in brain development and immune function show much greater variability. We also identified genes with random monoallelic expression and incomplete X chromosome inactivation. By capturing these diverse patterns, ASPEN provides a clearer view of the dynamic regulatory mechanisms that shape gene expression.

## Introduction

F1 hybrids, the offspring of inbred parental strains, provide a powerful model for identifying the genetic influences that shape gene regulation. By combining a controlled genetic background within a shared nuclear environment, F1 hybrids enable dissection of cis- and trans-regulatory contributions to expression variation. These hybrids have been used to uncover core principles of transcriptional regulation and epigenetic processes, including parent-of-origin effects [1–3], X-chromosome inactivation [4,5], cis-regulatory evolution [6,7], and the molecular basis of hybrid vigor [2,9]. Combined with high-throughput sequencing, this approach has been applied across taxa, from model mammals (e.g., mice [8,9]) and insects (e.g., *Drosophila* [10–12]) to plants (e.g., *Arabidopsis* [13–15]) and fungi [16–18].

The integration of F1 hybrid studies with single cell genomics has enabled resolution of phenomena such as transcriptional bursting [19,20], incomplete X inactivation [21,22], and dynamic cis-regulation across lineage differentiation [29]. Several analytical frameworks have been developed to assess allelic changes using single-cell RNA-seq data. These include *Airpart*, which uses a hierarchical Bayesian model to estimate cell type-specific allelic imbalances [23]; *scDALI*, which implements a generalized linear mixed model to detect variations in ASE across continuous trajectories [24]; and *SCALE*, which compares transcriptional kinetics between alleles within a hierarchical Bayesian framework [19]. Complementary approaches have been developed for allelic analysis in genetically heterogeneous or outbred populations; for example, *DAESC* uses random effects modeling to study allelic usage in humans [25]. While these tools have advanced the field, challenges remain in accurately detecting regulatory changes, particularly given the sparsity and noise inherent to single-cell data.

To address these limitations, we introduce ASPEN, a flexible statistical method for modeling allelic imbalance and variance in single-cell transcriptomic data from F1 hybrids. ASPEN combines a sensitive allele-specific quantification pipeline with a moderated beta-binomial model that incorporates adaptive shrinkage to stabilize dispersion estimates, enabling robust detection of allele-specific expression in droplet-based scRNA-seq. Applying ASPEN to mouse brain organoids and T cells, we uncover dynamic cis-regulatory changes during cellular differentiation, random monoallelic expression, and incomplete dosage compensation in X-linked genes.

## Results and discussion

### Regulatory control, not technical noise, underlies low allelic variation

**Fig 1:**
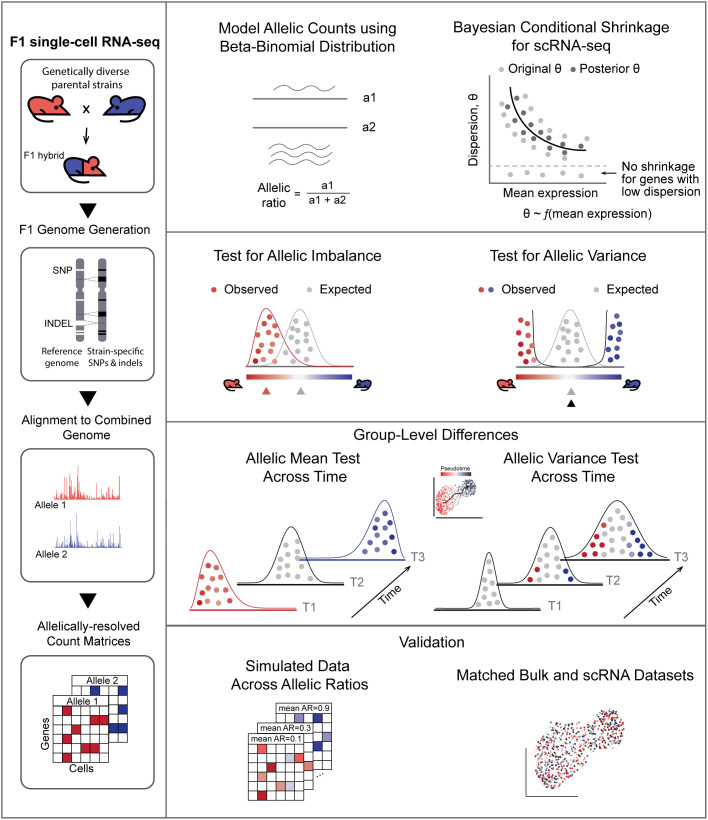
Overview of ASPEN. LHS: Raw sequencing reads are mapped to a combined genome created by concatenating the two parental genomes. The C57BL/6J (GRCm38) genome serves as the reference, while strain-specific genomes are produced by integrating variant information (SNPs and indels) into the Bl6 genome. RHS: ASPEN employs the Bayesian shrinkage method to stabilize dispersion estimates across genes with varying expression levels. Using weighted log-likelihood, original dispersion estimates are shrunk toward the common trend dispersion — the expected level of variation for genes exhibiting similar expression levels. The posterior (shrunken) dispersion estimates facilitate the detection of state-specific and differential changes in the allelic ratio distribution.

Analysis of single-cell allele-specific expression (scASE) data, particularly the high sparsity of droplet-based data such as 10x Chromium, is challenging due to high technical noise which can obscure true biological signal [26]. To address this, we implemented a highly sensitive mapping pipeline with a moderated beta-binomial model that features conditional shrinkage to accurately detect allele-specific gene regulation using single-cell data (Fig 1).

We first implemented a stringent read-mapping pipeline that integrates both single nucleotide polymorphisms (SNPs) and small insertions/deletions (indels) to align reads against custom F1 hybrid mouse genome assemblies. We created custom assemblies for common F1 crosses between the reference *C57BL/6J* (Bl6) strain and *CAST/Ei* (Cast), *MOLF/Ei* (Molf) *PWK/Ph* (Pwk),* and SPRET/Ei* (Spret) from the Hybrid Mouse Diversity Panel. By incorporating both SNPs and indels in the genome references, read mapping was improved by ~10% compared to using SNPs alone (S1 Fig). We only retained uniquely mapped reads for unambiguous allelic assignment as the incorporation of multi-mapping reads to increase read coverage reduced the number of allelic imbalance genes identified (S1 Appendix). Across four widely used mouse hybrid crosses, this strategy enabled the resolution of 20–38% of total sequencing reads. Due to genome quality, most aligned reads mapped to the reference genome (52–56%). ASPEN’s allelic imbalance test accounts for this mapping bias by adjusting the expected background ratio.

To improve statistical power of resolving allelic counts while accounting for data sparsity, we used a moderated beta-binomial modeling framework with empirical Bayes shrinkage of dispersion parameters. Allelic variance was parameterized as dispersion by quantifying the deviation around the beta-binomial mean allele ratio. Shrinkage was applied across the transcriptome, stabilizing dispersion estimates by borrowing information across genes while still accommodating gene-specific departures from the global trend, thereby reducing technical noise [27,28]. This approach is conceptually similar to that used in the differential gene expression analyses of bulk RNA-seq data [29,30].

**Fig 2:**
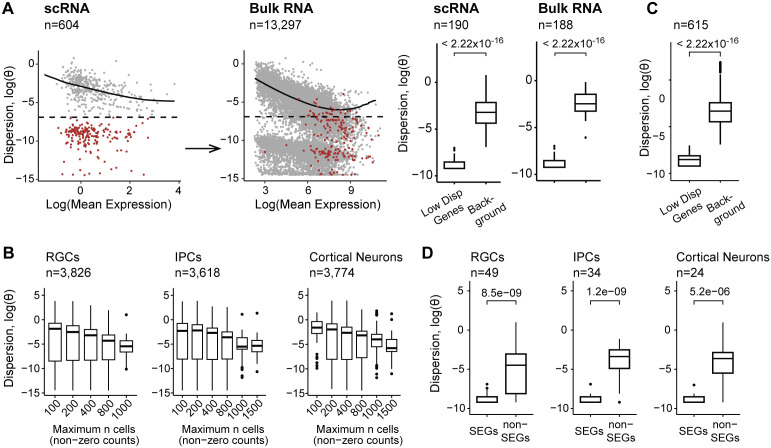
ASPEN performs adaptive dispersion modelling for single cell allelic data. (A) Evaluating the concordance of low dispersion for genes in matched bulk and single-cell data. Genes with θ < 0.001 in the scRNA counts are marked in red (left panel). The same genes are indicated in red based on the dispersion estimation in the bulk RNA-seq data (right panel). Comparisons were conducted using T-cell data collected on day 7 after LCMV infection. Boxplots illustrate the mean dispersion estimation between lowly dispersed genes and the background, a set of genes matched by gene expression (two-sided Wilcoxon rank-sum test). (B) Boxplots showing a reduction in variation levels as the number of available (non-zero count) cells increases: RGCs – H-statistic = 216.55, p < 2.22 $\times \;{\text{10}}^{-\text{16}}$, IPCs – H-statistic = 177.42, p < 2.22 $\times \;{\text{10}}^{-\text{16}}$, Cortical neurons – H-statistic = 160.54, p < 2.22 $\times \;{\text{10}}^{-\text{16}}$, Kruskal-Wallis rank sum test). (C) Low variation levels are not driven by gene expression or limited number of cells. Dispersion estimation comparison between genes with low dispersion (θ < 0.001) and background, genes matched by cells and reads (two-sided Wilcoxon rank-sum test). (D) SEGs demonstrate persistent low dispersion levels when compared with non-SEGs (two-sided Wilcoxon rank-sum test). Background (non-SEGs) is matched to SEGs by gene expression and number of detected cells.

We observed a set of low variance outlier genes that clustered distinctly below the modeled relationship between allelic dispersion and gene expression, despite meeting minimal read coverage threshold (Fig 2A). We hypothesized that these genes, which exhibit consistently low allelic dispersion, represent loci under stringent cis-regulatory control to maintain stable expression across cells. In such cases, overshrinkage could artificially inflate dispersion estimates and mask biologically relevant signals. To assess this, we compared allelic dispersion profiles between single-cell and bulk RNA-seq datasets generated from activated CD8 + T cells (day 7 post-infection) in *C57BL/6J* × *SPRET/Ei* (Bl6 x Spret) F1 hybrids [31,32]. Genes with low dispersion in single-cell data (θ < 0.005) maintained highly stable allelic ratios and exhibited robust expression in bulk RNA-seq (Wilcoxon Z = –8.23, *p* < 2.2 × 10 ⁻¹⁶; Fig 2A).

Functional enrichment analysis revealed the underdispersed genes were significantly enriched in housekeeping processes (FDR < 0.1; S2A Fig), suggesting these pathways may be subject to stronger constraint and tightly coordinated expression. To assess whether the observed low dispersion was the result of sampling noise from low cell numbers and low expression, we randomly subsampled cells to match the distribution of average read counts and the number of cells with non-zero expression in low-dispersion genes (Fig 2B). In contrast, the matched background genes exhibited much greater dispersion, indicating that the low dispersion observed was driven by biological effects rather than sampling noise (*p* < 2.22 × 10 ⁻^16^, two-sided Wilcoxon test, Fig 2C).

We further compared our dispersion estimates to a list of stably expressed genes (SEGs) identified through integrative analyses across multiple tissues and conditions [33], providing an independent benchmark for assessing gene expression stability. Genes classified as SEGs had lower allelic dispersion in our single-cell dataset compared to non-SEGs at matched expression level and cell coverage (median ∆θ = 0.2, Wilcoxon p ≤ 5.2 × 10 ⁻^6^; Fig 2D, S2B Fig). SEGs were also generally more enriched among our low-variance genes (Fisher’s exact, OR = 1.3, *p =* 0.04), suggesting these genes were under stronger cis-regulatory control.

In summary, we identified a set of genes with low allelic variances in scRNA-seq datasets, enriched for core housekeeping functions. Their variance remained low even at high expression levels, consistent with stable regulatory control.

### ASPEN robustly detects allelic imbalance in single-cell data

**Fig 3:**
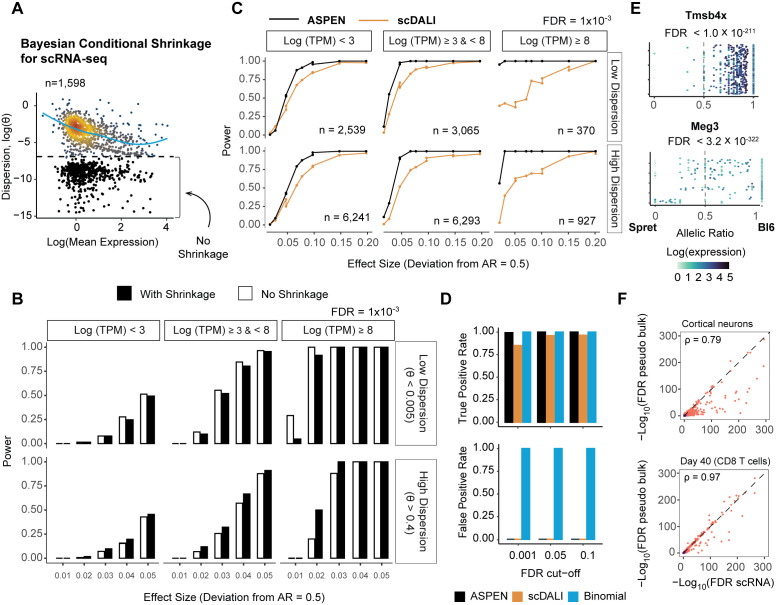
Evaluation and comparison of ASPEN’s performance in detecting ASE imbalance (A) Modeling the relationship between allelic ratio dispersion and mean gene expression revealed a population of genes with persistent low allelic dispersion in T-cell data from male Bl6 × Spret, combining all cell states. The dashed line separates genes with low variation (in black), which are excluded from the shrinkage procedure. (B) Evaluating Bayesian (shrinkage applied to all genes) and non-Bayesian (no shrinkage applied) strategies for testing allelic imbalance. Using simulated data, power was estimated as the fraction of genes detected as allelically imbalanced at FDR = 0.001, stratified by dispersion level: low (θ < 0.005) and high (θ > 0.4); and gene expression levels – low (Mean(logTPM) < 3), medium (mean(logTPM) ≥ 3 & Mean(logTPM) < 8) and high (Mean(logTPM) ≥ 8). (C) Performance comparison between ASPEN and scDALI using simulated data. Line plots illustrate TPRs calculated at FDR = 0.001 for genes categorized by gene expression and allelic dispersion levels (low dispersion – θ < 0.005, medium – θ ≥ 0.005 & θ < 0.4, and high – θ ≥ 0.4) across groups with varying degrees of deviation from the mean AR = 0.5. Only datasets simulated with a mean AR of 0.5 ± 0.2 are displayed. No performance differences were identified for groups with deviations from balanced allelic expression > 0.2. (D) Comparing performance in detecting allelic imbalance. TPRs are calculated based on the results from the simulated data with mean AR = 0.4. (E) Examples of the allelic ratio distributions of X-linked genes (*Tmsb4x*) and imprinted genes (*Meg3*). (F) Correlation of ASPEN-log_10_FDR values generated by running ASPEN mean test on the single-cell counts (x-axis) and pseudobulked counts (y-axis). Analyses were conducted in mouse cortical neurons (n = 972) from female Bl6 × Cast F1 hybrids and in CD8^+^ T cells at day 40 (n = 768) following LCMV infection in male Bl6 × Spret F1 hybrids.

Based on our analyses above, we reasoned that genes with low dispersion likely reflect biological constraint. For such genes, shrinkage, normally used to stabilise noisy genes, would instead inflate their dispersion and reduce the statistical power to detect imbalance. Therefore, ASPEN does not apply shrinkage to those genes with intrinsically low dispersion (Fig 3A**, Methods**). Following estimating dispersion parameters, ASPEN tests for allelic imbalance using a likelihood ratio test that compares a null model, which assumes a mean corresponding to empirically determined allelic balance, with an alternative model that estimates the mean allelic ratio directly using maximum likelihood estimation (MLE).

To validate this framework, we performed extensive simulations across a range of gene expression levels, overdispersion parameters, and allelic levels. For realistic simulations, parameters of the beta-binomial model were estimated from a mouse brain organoid dataset [34].

ASPEN consistently demonstrated high sensitivity for detecting allelic imbalance with low false positives (Fig 3B, S1 Table in S2 Appendix). For low-dispersion genes, exemption from shrinkage improved sensitivity by up to 22%. In contrast, genes with high allelic dispersion benefited from shrinkage, which enhanced detection accuracy by up to 30%. We benchmarked ASPEN against scDALI [24], a method that models ASE via generalized linear mixed models, and found that ASPEN detected up to ~30% more imbalanced genes across simulated datasets, with no detectable increase in false positives among allelically balanced controls (Fig 3C, S3A Fig). We also compared ASPEN’s method to the binomial test. As expected, because the binomial test does not account for overdispersion, the binomial test showed extremely high false positive rates in single cell droplet-based data (Fig 3D). ASPEN runtimes for varying numbers of genes and cells are reported in S3B Fig.

We further assessed ASPEN’s ability to accurately resolve allele-specific expression using monoallelically expressed genes. In brain organoids derived from male Bl6 x Spret F1 hybrids, ASPEN reliably identified 100% of expressed X-linked genes (43/43) as monoallelic. Additionally, all known imprinted loci, including *Meg3* and *Peg3*, were correctly recovered (n = 10; Fig 3E, S2 Table in S2 Appendix), consistent with the ability to accurately resolve allele-specific expression.

We compared single-cell and bulk-level expression data. We aggregated scRNA-seq data into pseudobulk profiles across three major brain organoid subpopulations. ASPEN’s single-cell allelic imbalance calls showed strong concordance with pseudobulk-derived results, with 74–82% overlap in brain organoids and 77–93% agreement in T cells across different activation states (Fig 3F, S3C Fig). The slightly lower concordance in organoids may reflect a higher degree of cell-to-cell heterogeneity in allelic expression. This is consistent with prior observations of increased random monoallelic expression during embryonic stem cell differentiation [39] (S3D Fig).

We applied ASPEN to distinguish allelic imbalance in T cells from Bl6 x Spret [32], identifying significant imbalance in 491 out of 809 expressed genes, including canonical markers of T-cell activation (*Cd69*), effector differentiation (*Gzmb*, *Ifng*), and memory formation (*Tcf7*, *Il7r*) (FDR  <  0.05, S3 Table in S2 Appendix, S3E Fig). To explore potential regulatory mechanisms, we assessed whether allelic bias was associated with transcription factor (TF) motif differences. We used scATAC data from T-cells on day 7 post-LCMV infection from the Bl6 parental strain [32], focusing on genes with strong allelic bias (ASPEN-mean FDR < 0.05, |log2FC| ≥ 1). For each parental haplotype, we calculated motif enrichment scores in promoter regions, comparing log odds scores for motifs significant on both alleles (*p* <$\;\text{1}\times {\text{10}}^{-\text{4}}$). Across 53 genes and 312 motifs, we observed a mild correlation (ρ=0.2) between allelic bias and allele-specific TF motif enrichment (S3F Fig). Integrating chromatin data from F1 hybrids would enable more direct mechanistic inference.

In summary, ASPEN showed strong concordance between single-cell and bulk-level allelic imbalance calls and recovered expected monoallelic and imprinted genes. It outperformed alternative methods in sensitivity and false positive control.

### Beyond imbalance, modeling allelic variance

**Fig 4:**
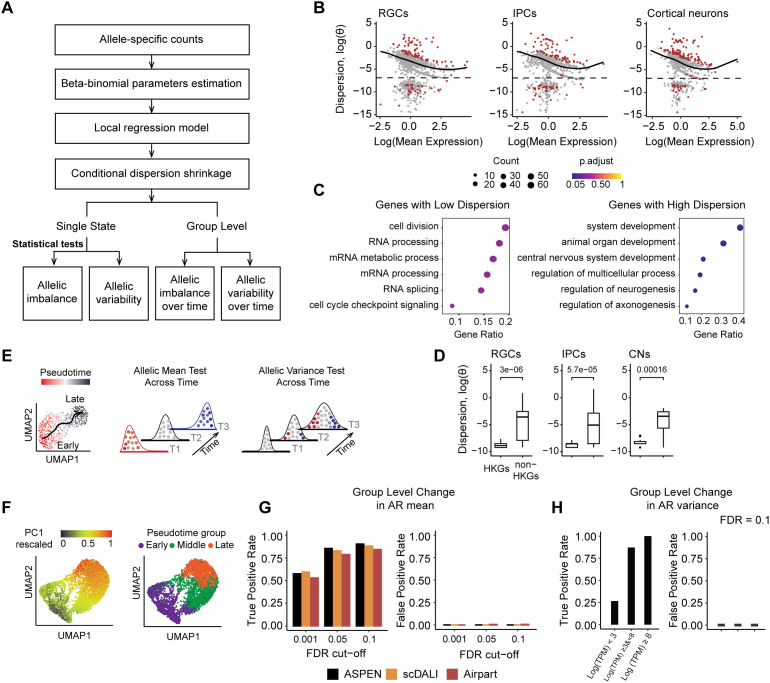
Detect allelic variation and differential changes in ASE with ASPEN. (A) Overview of the ASPEN analytical workflow. The main analysis steps involve the initial estimation of the allelic count distribution parameters, modeling allelic count dispersion as a function of gene expression, and conditional shrinkage toward the common dispersion estimates. (B) Results of the ASPEN var test in brain organoids data. Genes with significant deviation from expected variation levels are in red (FDR < 0.05): cortical neurons n = 140 (out of 973), IPCs n = 117 (out of 857) and RGCs n = 108 (out of 1,078). Genes below the dashed line were excluded from the trend modelling. (C) Top six Gene Ontology (GO) functional terms enriched in lowly dispersed genes (below the trend line) and highly dispersed genes (above the trend line). (D) Genes with housekeeping function in ESCs are lowly dispersion in different brain organoids cell types in female Bl6 × Cast F1 hybrids (RGCs: n = 26, IPCs: n = 20, CNs: n = 20, two-sided Wilcoxon rank-sum test). (E) Schematic representation of the ASPEN strategy used to detect changes in allelic mean or variance over time. (F) UMAP visualization of the simulated dataset used for testing. The cells are colored based on the rescaled PC1 coordinates representing temporal ordering (left) and by the cell assignments to discrete pseudotime bins: early, middle, and late (created by dividing the PC1 vector into tertiles) (right). (G) *Group-mean* test performance metrics. Test is applied to data simulated with group changes in allelic mean (0.45, 0.5, 0.55) vs. control (0.5, 0.5, 0.5). (H) G*roup-var* test performance metrics across different gene expression thresholds: low – Mean(logTPM) < 3, medium - Mean(logTPM) ≥ 3 & < 8, high - Mean(logTPM) > 8. FDR = 0.1 was used for the evaluation. Data was simulated with changes in allelic variance (1 × 10^-3^, 0.05, 0.2) vs. control (1 × 10^-3^, 1 × 10^-3^, 1 × 10^-3^).

A key feature of ASPEN is its statistical framework to test for allelic over- and under- dispersion, enabling the identification of loci where allelic variance deviates from expression-level expectations (Fig 4A, S4 Fig, **Methods**). Reduced levels of allelic variance may reflect transcriptional precision, while increased variance can signal regulatory plasticity, stochastic gene expression, or context-dependent modulation of cis-regulatory inputs [35].

To detect such deviations, ASPEN first models the expected allelic dispersion as a function of gene expression, capturing the transcriptome-wide trend. It then evaluates whether individual genes significantly depart from this trend using an empirical permutation-based procedure applied to stabilized dispersion estimates (**Methods**). In female *C57BL/6J x CAST/Ei* (Bl6 x Cast) F1 hybrid brain organoids, we identified 232 out of 1,261 expressed genes whose allelic variance significantly deviated from the expectation (Fig 4B). Of these, 35% (83/232) were significantly underdispersed, while 65% (149/232) displayed significant over dispersion.

Genes with reduced allelic variance were linked to core cellular processes, including RNA splicing, mRNA metabolism, and cell cycle regulation (FDR < 0.5; Fig 4C, left panel), consistent with prior observations. Many have reported housekeeping function in ESCs [36], suggesting that allelic stability is selectively maintained in genes essential for cell function (Fisher’s exact OR = 1.42, *p* = 0.02, Fig 4D, S5A Fig). By contrast, overdispersed genes were enriched for neurodevelopmental pathways, including glial differentiation (*Sox4, Olig1, Ptn, Nfib*), axonogenesis (*Dcx, Pac3, Map1b, Nefm*), neurogenesis (*Vim, Nrp1, Sox11, Syt4*), and CNS development (*Tubb2a, Cenpf, Fabp7, Neurog2*) (FDR < 0.05; Fig 4C, right panel).

To capture dynamic regulatory shifts during differentiation, ASPEN implements two likelihood ratio tests: one to detect temporal changes in allelic mean (*group-mean*) and another to test for temporal shifts in allelic variance (*group-var*) (Fig 4A, 4E). In both cases, a null model assumes a shared set of beta-binomial parameters across timepoints, whereas the alternative model allows group-specific parameters. For the *group-mean* test, the mean is allowed to vary across the groups to capture distribution changes, while in *group-var*, the mean is fixed to assess differential changes in variance (Fig 4E).

To benchmark the performance of ASPEN for group analyses, we simulated single-cell data along a pseudo-time axis (**Methods**, Fig 4F). We divided the pseudotime vector into discrete equal-sized bins and cells were grouped into early, intermediate, and late stages. Simulations modeled: 1) group-level changes in allelic mean, 2) group-level changes in allelic variance, and 3) a null model with stable parameters (S5B Fig).

ASPEN *group-mean* outperformed scDALI-Het and AirPart in detecting mean ASE differences with comparable specificity (sensitivity: 90%, 88%, 84%, respectively; specificity: 100%; FDR = 0.1(Fig 4, S5C Fig, S4 Table in S2 Appendix). ASPEN’s *group-var* test yielded 64% sensitivity and 100% specificity at FDR = 0.1 across all genes but increasing to > 87% sensitivity for more highly expressed genes (~60% of genes) (Fig 4H, S5D, S5 Table in S2 Appendix).

Applying ASPEN to early neurogenesis in female Bl6 x Cast F1 hybrids revealed 162 genes with significant changes in mean allelic expression and 306 with differential variance, independent of total expression changes (S5E Fig; S6 and S7 Tables in S2 Appendix). These included regulatory kinases (*Csnk1a1*), chromatin and transcriptional modulators (*Pbx1, Hmgb1, Fos*), and transcriptional co-regulators (*Taf1d, Ccna, Trim28r1, Eid1*). Notably, early progenitor populations exhibited the lowest variance (θ = 0.07 ± 0.01), which marginally increased in intermediate progenitors (θ = 0.09 ± 0.01) and differentiated cortical neurons (θ = 0.10 ± 0.02). This pattern was consistent with a reported increase in cellular heterogeneity during lineage commitment [37].

Eight genes with differential variance, including *Ankrd11, Eif3g, Nrxn1*, were high-confidence autism risk genes (SFARI database) [38]. Additionally, five genes exhibiting variable allelic expression (*Eif3h, Lars, Oaz1, Selenok, Hsp90aa1*) have been implicated in anatomical brain phenotypes based on mouse knockout models [39] (S5F Fig). We propose that temporal modulation of variance may reflect regulatory mechanisms that modulate gene expression programs during development and could be perturbed in disease states.

To summarize, ASPEN tests whether allelic variance at each gene departs from the transcriptome-wide expectation. This revealed a stabilization of variance in genes essential for core functions, highlighting regulatory flexibility in neurodevelopmental and disease-linked loci during differentiation.

### Detection of random and incomplete X inactivation

**Fig 5:**
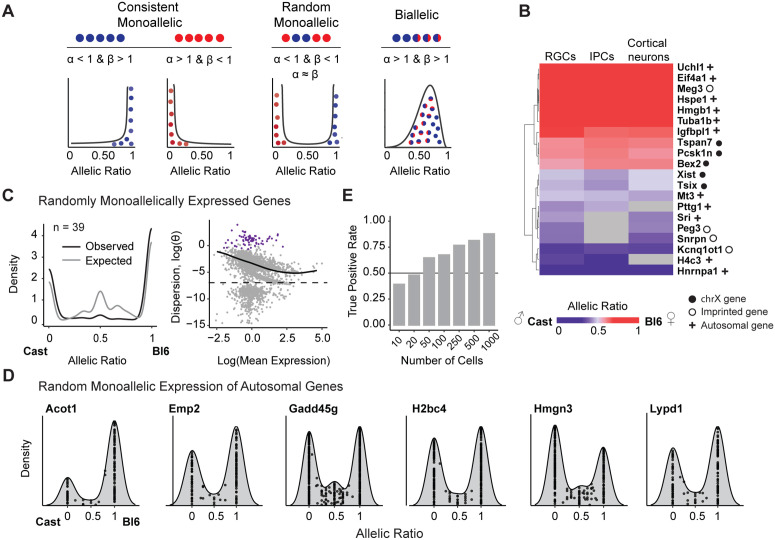
Monoallelic expression patterns in early neurogenesis. (A) Scenarios where allelic variance deviates from expected levels and the corresponding beta-binomial distribution shape parametersα and β. (B) The heatmap displays the mean allelic ratios across cells for genes with monoallelic expression. Genes were classified as monoallelic if their estimated beta-binomial α or β values were less than one. Results are from brain organoid data derived from female Bl6 × Cast hybrids using three cell types from the early neurodevelopmental pathway. Genes with allelic imbalance detected across all three cell types, with a mean expression above 1 in at least one, were selected for plotting (n = 18). Rows were hierarchically clustered using complete linkages. Genes without sufficient coverage in one of the cell types are indicated in grey. (C) Evaluating the expected versus observed dispersion distributions of genes with random monoallelic expression (RME) (total n = 39). The expected allelic ratio is calculated as the ratio of the simulated reference allele count to the total count. The expected gene set comprises genes without evidence of monoallelic expression, matched by expression to RME genes. The departure of the RME genes (in purple) from the common dispersion trend is visualized in the mean-variance plot. (D) Allelic ratio distribution plots for a selection of autosomal RME genes. (E) RME detection rates were low in the data simulated with a limited number of cells (n ≤ 20). RME simulation was performed with varying cell numbers (n = 10, 20, 50, 100, 250, 500, 1000).

To identify genes with monoallelic expression, we used ASPEN’s estimation of beta-binomial shape parameters α and β. These parameters represent the support for expression from each allele, with values below 1 indicating cells showing monoallelic expression (Fig 5A). When both α and β are below 1, cells express either one allele or the other, producing a bimodal allelic distribution consistent with random monoallelic expression (RME), in which a single allele is stochastically expressed in individual cells [40–43] (Fig 5A).

Among 1,385 genes expressed in neuronal cells from female Bl6 x Cast F1 hybrids, 72 demonstrated monoallelic patterns, defined by extreme skew in the beta-binomial shape parameters, wherein either α or β was less than 1 (Fig 5A-5B, S8 Table in S2 Appendix). Filtering based on these parameters identified canonical imprinted genes, including *Meg3*, *Peg3*, *Kcnq1ot1*, *Rian*, and *Snrpn,* validating the approach (Fig 5, S6A Fig).

To identify genes showing random monoallelic expression, we required both α and β < 1, with a difference between the parameters of less than 0.5 and further confirmed these candidates using the ASPEN variance test. We identified 39 high-confidence RME genes (Fig 5C). Of these, 27 were X-linked, consistent with random X-inactivation dynamics in females. Five genes (*Bex2*, *Ndufb11*, *Pcsk1n*, *Sh3bgrl*, *Uba1*) were predominantly monoallelic in expression, yet they possessed a small proportion of cells (15–33%) displaying biallelic expression, consistent with incomplete X inactivation (S6B Fig). Twelve RME genes were autosomal (Fig 5D; S6C Fig). These included *Gadd45g* and *H2bc4,* which we identify here as candidate RME genes.

We examined whether expression level and cell numbers could have biased our results. RME genes were not more lowly expressed than non-RME genes (D = 0.182, p = 0.08, two-sided K-S test) but they were detected in fewer cells. To assess the impact of cell number level on ASPEN results, we simulated RME genes with different cell numbers, matching the distribution observed in real data. ASPEN achieved a sensitivity of 81.3% in simulations with a comparable number of cells (n = 500) to the median cell numbers for RME genes detected by ASPEN in real data (n = 526) (Fig 5E, S6D-S6E Figs.). Notably, most autosomal RME genes recovered by ASPEN have been independently validated by clonal expansion followed by bulk RNA-seq, demonstrating ASPEN’s ability to detect RME *in vivo* (S6F Fig) [37,44,45].

### ASPEN detects increased cis-regulatory control at housekeeping genes and key regulators of effector fate

**Fig 6:**
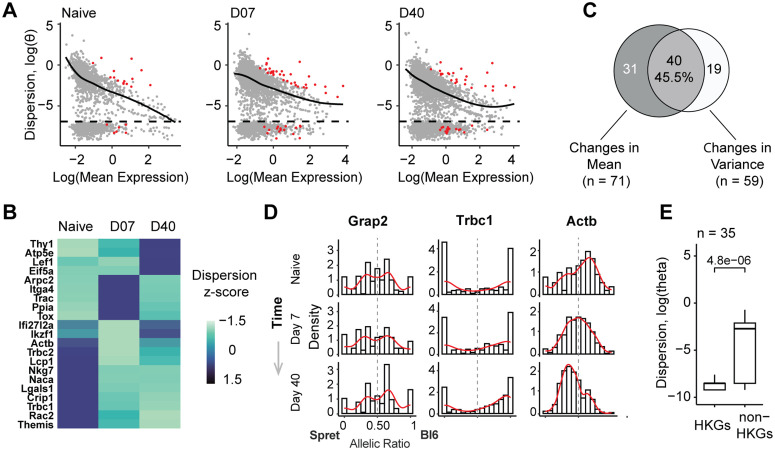
Evaluating allelic changes during T-cell activation. (A) Genes with significant deviations from the expected dispersion levels (in red) determined by the ASPEN var test (FDR < 0.05): Naïve n = 21, day 7 n = 50, day 40 n = 41. (B) Genes with housekeeping function had persistently low dispersion compared to background, matched by gene expression and number of detected cells (two-sided Wilcoxon rank-sum test). (C) Heatmap of the dispersion z-scores for the genes with ASPEN FDR var < 0.05 in at least one cell state (n = 21). Z-scores represent the empirical dispersion estimates for each gene scaled by column. (D) Venn diagram showing the overlap between the genes with dynamic changes in mean (ASPEN group-mean: FDR < 0.05) and variance (ASPEN group-var: FDR < 0.05). (E) Examples of differential changes in allelic distribution during T-cell activation. ASPEN tests categorize genes into three groups: those changing only in variance and not in mean (*Naca*, FDR group-var = 3.27 × 10^-8^, FDR group-mean = 1); those with changes in both mean AR and variance (*Trbc1*, FDR group-var = 2.57 × 10^-3^, FDR group-mean < 1.4 × 10^-96^); and those changing only in mean and not in variance (*Actb*, FDR group-var = 1, FDR group-mean < 1.4 × 10^-96^).

Finally, we applied ASPEN to dissect the allelic regulatory dynamics during T-cell activation in acute viral infection, during which naïve CD8 + T cells transition through effector and memory states. Among 809 expressed genes, ASPEN identified 90 loci with allelic variance that deviated significantly from expression-level expectations (FDR < 0.05, Fig 6A).

Consistent with observations in brain organoids, underdispersed genes in T cells were also enriched for broadly conserved cellular processes (S7A Fig), including housekeeping function (OR = 2.33, *p* = 2 × 10 ⁻^3^, Fisher’s exact test) (Fig 6B, S7B Fig). Furthermore, genes critical for CD8 + T cell clonal expansion and effector differentiation, such as *Il2ra* and *Cd8a*, exhibited significantly lower allelic variance relative to non-critical genes at matched expression level (Wilcoxon Z = –5.54, *p* = 3.04 × 10⁻⁸; S7C Fig) [46]. In contrast, overdispersed genes were associated with immune-specific functions. These included pathways related to immune system processes (*Trac, Tox, Nkg7, Ccl5*), lymphocyte activation (*Lgals1, Actb, Themis, Lef1*), and leukocyte signaling (*Myo1f, Klrd1, Thy1, Ccnd3*) (S7A Fig).

Using ASPEN’s group-var test, we identified a shift in allelic variance over the course of T-cell activation. Naïve cells exhibited the highest allelic variance (θ = 0.12 ± 0.04), which progressively declined at day 7 post-infection (θ = 0.05 ± 0.01) and further decreased in memory cells at day 40 (θ = 0.02 ± 0.01) (Fig 6C, S7D Fig). Among 119 differentially expressed genes, ASPEN identified significant temporal changes in allelic mean ratios for 74 genes (62%) and differences in allelic variance for 67 genes (56%), with 55 genes showing coordinated changes in both parameters (FDR < 0.05, Fig 6D, S9 Table in S2 Appendix).

Genes exhibiting differential allelic regulation showed distinct patterns of allelic expression (Fig 6E, S7E Fig). For example, *Naca*, nascent polypeptide-associated complex subunit alpha involved in T cell proliferation, showed changes in variance but not in allelic ratio (Fig 6E). In contrast, *Actb* demonstrated a shift in allelic preference without concomitant changes in variance, while *Trbc1* transitioned from biallelic to maternally biased expression while maintaining stable total transcript levels (Fig 6E).

Together, these examples highlight the diversity of expression dynamics. Allelic variance decreased as cells were activated, consistent with increased transcriptional control with functional specialization. Core effector genes for T cell activation exhibited low variances independent of expression level, suggestive of regulatory control.

## Conclusion

Allelic expression dynamics offer a powerful lens for dissecting cis-regulatory mechanisms, yet their detection in single-cell data has been limited by technical noise and data sparsity. ASPEN addresses these challenges through a sensitive mapping pipeline, moderated beta-binomial modeling, and adaptive shrinkage, achieving up to 30% greater sensitivity than existing methods while maintaining stringent false discovery control.

Beyond mean allelic ratios, ASPEN quantifies allelic variance—a dimension of cis-regulation that has remained largely unexplored at single-cell resolution. Our analyses reveal reduced variance in housekeeping and essential genes, consistent with tight regulatory control, and elevated variance in neurodevelopmental and immune-related genes, indicative of stochastic or context-dependent regulation. ASPEN further enables detection of random monoallelic expression and incomplete X inactivation, extending these insights to rare and postmitotic cell types where clonal expansion approaches are infeasible. Together, these results establish allelic variance as a key regulatory dimension and demonstrate that ASPEN provides a robust framework for uncovering the dynamic cis-regulatory programs that shape gene expression across cellular states.

## Methods

We generated F1 alignment indices for four common mouse hybrid strains: *C57BL/6J* (Bl6) *× Mus Castaneus (CAST/Ei)* (Cast), Bl6 × *Mus musculus molossinus (MOLF/Ei)* (Molf), Bl6 × *Mus musculus musculus (PWK/Ph)* (Pwk), and *C57BL*/*6J × Mus Spretus (SPRET/Ei)* (Spret). Reads from hybrid animals were aligned to their parental combined genomes. Only reads that could be allelically resolved were used in gene expression quantitation.

### Combined genome generation

We generated combined genomes containing two sets of chromosomes for each genetic background, which allowed for unambiguous assignment of allelic reads. Variant call files (release 5) for four wild-derived mouse strains (*CAST/Ei,* *MOLF/Ei*, *PWK/Ph*, and *SPRET/Ei*) were downloaded from the Mouse Genome Project [47]. Strain-specific genomes were created using high-quality calls (filter set to PASS, GT = “1/1”) and incorporating (i) only SNPs and (ii) both SNPs and insertions and deletions (indels) into the reference Bl6 genome (GRCm38, release 68) with BCFtools consensus (v1.12) [48]. Each genome was concatenated with the reference genome to produce combined genomes for each mouse strain. The chain files from the BCFtools were used to create strain-specific gene annotations by converting Bl6 gene annotations (Ensembl release 102) via UCSC liftOver [49]. Combined genome index files were generated using STAR (v2.7.6a) [50].

### Beta-binomial modeling of allelic counts

To quantify allele-specific expression, ASPEN requires as input two matrices for each cell and gene: the number of reads mapping to the reference allele (${\text{m}}_{\text{j}})$ and the total read count (${\text{n}}_{\text{j}}$), where i indexes genes and j indexes cells. For each gene, ASE is modeled using a beta-binomial distribution, parameterized by a mean allelic ratio ($\mu_{\text{i}}$) and a dispersion parameter ($\theta_{\text{i}}$), which together capture the probability and variability of observing reference allele counts across cells. The likelihood of observing ${\text{m}}_{\text{j}}$ reference reads out of ${\text{n}}_{\text{j}}$ total reads for gene *i* in cell *j* is given by:


$$\begin{array}{c} \text{P}\left({{\text{m}}_{\text{ij}}|\text{n}}_{\text{ij}},\;\alpha_{\text{i}},\;\beta_{\text{i}}\right)=(\begin{array}{c} {\;\text{n}}_{\text{ij}}\;\\ {\text{m}}_{\text{ij}} \end{array})\frac{\text{Beta}\left(\alpha \;+\;{\text{m}}_{\text{ij}},\;\beta_{\text{i}}\;+\;{\text{n}}_{\text{ij}}\;-\;{\text{m}}_{\text{ij}}\right)}{\text{Beta}\left(\alpha ,\;\beta \right)},\;\alpha_{\text{i}},\;\beta_{\text{i}}\;>\;\text{0}, \end{array}$$

where $\alpha_{\text{i}}$ and $\beta_{\text{i}}$ are the shape parameters of the beta prior. These are related to the mean and dispersion by:

${\hat{\mu }}_{\text{i}}\;=\frac{\alpha_{\text{i}}}{\alpha_{\text{i}}+\beta_{\text{i}}}$ and ${\hat{\theta }}_{\text{i}}\;=\frac{\text{1}}{\alpha_{\text{i}}\;+\;\beta_{\text{i}}}.$

Here, ${\hat{\mu }}_{\text{i}}$ represents the average allelic ratio for gene *i*. and ${\hat{\theta }}_{\text{i}}$ reflects the overdispersion or variability in allelic ratios across cells beyond binomial sampling noise [51].

Parameter estimation is performed by maximizing the likelihood function:


$$\text{L}\;=\;(\begin{array}{c} \;{\text{n}}_{\text{ij}}\;\\ {\text{m}}_{\text{ij}} \end{array})\;\frac{\Gamma (\alpha_{\text{i}}+\beta_{\text{i}})}{\Gamma \left(\alpha_{\text{i}}\right)\;\Gamma (\beta_{\text{i}})}\;\frac{\Gamma (\alpha_{\text{i}}+{\text{m}}_{\text{ij}})\;\Gamma ({\text{n}}_{\text{ij}}-{\text{m}}_{\text{ij}}+\;\beta_{\text{i}})}{\Gamma (\alpha_{\text{i}}+\beta_{\text{i}}+{\;\text{n}}_{\text{ij}})}.$$

Maximum likelihood estimation (MLE) was performed with R (v4.3.1) [52] ‘optim’ function using BFGS quasi-Newton method with positive constraints on $\alpha_{i}$ and $\beta_{i}$.

### Modeling the relationship between allelic dispersion and mean expression

ASPEN moderates gene-wise dispersion estimates by leveraging the overall relationship between dispersion and mean expression across all genes to address sampling noise and technical variation. This information-sharing uses a weighted log-likelihood framework [33,34,48]:


$$\begin{array}{c} l_{\text{WL}}\left(\theta_{\text{i}}\right)\;=\;l\left({\hat{\theta }}_{\text{i}}\right)\;+\;{\delta \;l}_{\text{SH}}\left({\overset{―}{\theta }}_{\text{i}}\right), \end{array}$$

where $\begin{array}{c} l\left(\hat{\theta }\text{i}\right)\; \end{array}$ is the log-likelihood for the gene-specific dispersion estimate, $\begin{array}{c} l_{\text{SH}}\left({\overset{―}{\theta }}_{\text{i}}\right) \end{array}$ is the log-likelihood for the shared dispersion at matched expression level, and δ determines the relative influence of the shared trend.

To estimate the shared dispersion values, we model the dependence of gene-level dispersion, ${\hat{\theta }}_{\text{i}}$, as a function of gene expression (${\overset{―}{\text{n}}}_{\text{i}}$ the average total read count per gene) using local regression implemented in the locfit (v.1.5-9.8 [53]) R package:

$\begin{array}{c} \text{log}\left({\hat{\theta }}_{\text{i}}\right)=\;f\;\text{log}\left({\overset{―}{\text{n}}}_{\text{i}}\right)\;+\;\varepsilon_{\text{i}}, \end{array}$

where *f* captures the smoothed mean-variance relationship and $\varepsilon_{\text{i}}\;$ is the residual for gene *i*. All genes are included in the regression to define a representative mean-variance trend. The resulting fitted values ${\overset{―}{\theta }}_{\text{i}}$, represent the expected dispersion for genes expressed at similar levels.

### Bayesian shrinkage implementation

ASPEN applies empirical Bayes shrinkage to stabilize dispersion estimates by combining gene-specific information with the transcriptome-wide mean-variance trend. For each gene, the posterior (shrunken) dispersion is calculated as a weighted average of the individual and local trend estimates, with the weight parameter (δ) controlling the degree of shrinkage. To obtain the posterior (shrunken) dispersion for gene *i*, we modified the analytical solution which was derived for the double binomial distribution and extended to the beta-binomial distribution [48]:


$${\hat{\theta }}_{\text{WLi}}=\frac{\text{N}}{\text{N}-\text{1}}\frac{{\hat{\theta }}_{\text{i}}\;+\;\delta {\overset{―}{\theta }}_{\text{SHi}}}{\text{1}+\delta },$$

where ${\overset{―}{\theta }}_{\text{SHi}}$ is the local trend, obtained by local regression on log-dispersion versus log-expression, serving as a prior for each gene’s dispersion, ${\hat{\theta }}_{i}$ is the individual gene dispersion estimate, N is the effective degrees of freedom, and δ is weight given to the shared likelihood. ${\overset{―}{\theta }}_{\text{SHi}}$ is treated as prior for $\theta_{\text{i}}$ under a Bayesian hierarchical framework. To avoid overfitting, the shrinkage weight is adjusted by the factor N-K, where K is the number of fitted parameters, yielding $\delta =\;\frac{\delta }{\text{N}-\text{K}}$. Hyperparameters for shrinkage are determined by fitting a gamma distribution to the observed dispersions across genes using maximum likelihood. All optimization is performed in R (v4.3.1) using the BFGS method [52] s. Initial parameters were set to N = 20 and δ = 10. For all real and simulated data, we set δ = 50 and N = 30, as determined empirically from T cell data.

### Treatment of genes with low allelic variation

We applied selective shrinkage for genes with low dispersion estimates that pass the read count threshold (≥ 5 cells ≥5 UMIs) which are moderated towards the common trend. To detect these lowly variable genes, we plot estimated dispersion against the mean expression. We fit a smoothing function across all data points using the local regression residuals, $\varepsilon_{\text{i}\;}$:


$$\begin{array}{c} \varepsilon_{\text{i}\;}=\;{\text{log}(\hat{\theta }}_{\text{i}})\;-\;f\;\text{log}\left({\overset{―}{\text{n}}}_{\text{i}}\right). \end{array}$$

Low variance genes are the outliers that cluster distinctly from the model trend. To provide a more principled approach, we also applied the median absolute deviation-squared, ${\text{MAD}}^{\text{2}}$ statistic [54],


$${\text{MAD}}^{\text{2}}=({\text{median}\;\left(\left|\varepsilon_{\text{i}}-\text{median}(\varepsilon_{\text{i}})\right|\right))}^{\text{2}}$$

and defined lowly variable genes as those with $\varepsilon_{\text{i}}<\;{\text{MAD}}^{\text{2}}.$ Classification of lowly variable genes used the threshold of θ < 0.001 for the real data and θ < 0.005 for the simulated data.

We note that for allelic tests, the dispersion trend was modeled after excluding these lowly variable genes from Bayesian shrinkage and updating the shared estimates, ${\overset{―}{\theta }}_{\text{i}}$.

### Statistical framework for testing allelic imbalance

After estimating gene-specific beta-binomial parameters and obtaining shrunken dispersion values, ASPEN tests for allelic imbalance using a log-likelihood ratio test. For each gene, the null hypothesis (${\text{H}}_{\text{0}}$) assumes allelic ratios follow a beta-binomial distribution with a fixed mean (*x*), reflecting the expected background allelic ratio, and the estimated dispersion. The alternative hypothesis (${\text{H}}_{\text{1}}$) allows the mean allelic ratio (${\hat{\mu }}_{\text{i}}$) to be estimated from the data:


$${\text{H}}_{\text{0}}:\;\text{AR}\;\sim \;\text{BetaBinomial}\;\left({\text{m}}_{\text{ij}}|{\text{n}}_{\text{ij}},\;\mu =\text{x},\;{\hat{\theta }}_{\text{WLi}}\right),$$

$${\text{H}}_{\text{1}}:\;\text{AR}\;\sim \;\text{BetaBinomial}\;\left({\text{m}}_{\text{ij}}|{\text{n}}_{\text{ij}},\;\mu ={\hat{\mu }}_{\text{i}},\;{\hat{\theta }}_{\text{WLi}}\right),$$

where x is the fixed allelic ratio which is determined based on empirical background (except for simulated data where x = 0.5, representing theoretical allelic balance), ${\hat{\mu }}_{\text{i}}$ is the allelic ratio and $\;{\hat{\theta }}_{\text{WLi}}$ is the shrunken dispersion that are specific to each gene. Both null and alternative models are fit by maximum likelihood in R as above.

For low-variance genes, the empirical (unshrunken) dispersion estimate, ${\hat{\theta }}_{\text{i}}$ is used instead of the shrunken value (${\hat{\theta }}_{\text{WLi}}$). The fixed allelic ratio x is set to the global mean across all autosomal, non-imprinted genes passing quality filters, or to 0.5 in simulated data. A likelihood ratio test (LRT) is then performed, comparing the fit of the null and alternative models. Test statistics are evaluated using the $\chi^{\text{2}}$ distribution with one degree of freedom, and *p*-values are corrected for multiple testing using the false discovery rate (FDR) [55].

### Statistical framework for testing changes in allelic variance

For each gene, we quantified allelic variation in two steps. First, we establish the baseline level of expected variation (${\overset{―}{\theta }}_{\text{SHi}}$) using local regression to model the relationship between expression level and allelic variation across all genes. We then compared each gene’s observed stabilized dispersion (${\hat{\theta }}_{\text{WLi}}$) against this, holding the mean allelic ratio (${\hat{\mu }}_{\text{i}}$) constant. Both dispersion and mean parameters were estimated by MLE. By analyzing the fit of these models, the test distinguishes genes where ASE patterns vary between cells due to biological processes rather than technical noise.

To detect genes with significantly greater or lesser allelic variance than expected for their expression level, we performed a likelihood ratio test based on the following hypotheses:


$${\text{H}}_{\text{0}}:\;\text{AR}\;\sim \;\text{BetaBinomial}\left({\text{m}}_{\text{ij}}|{\;\text{n}}_{\text{ij}},\;\mu ={\hat{\mu }}_{\text{i}},\;\theta ={\overset{―}{\theta }}_{\text{SHi}}\right),$$

$${\text{H}}_{\text{1}}:\;\text{AR}\;\sim \;\text{BetaBinomial}\left({\text{m}}_{\text{ij}}|{\;\text{n}}_{\text{ij}},\;\mu ={\hat{\mu }}_{\text{i}},\;\theta =\;{\hat{\theta }}_{\text{WLi}}\right),$$

where ${\overset{―}{\theta }}_{\text{SHi}}$ is the predicted baseline dispersion and ${\hat{\theta }}_{\text{WLi}}$ is the gene’s stabilized (shrunken) dispersion. Shrinkage is applied to all genes meeting minimum filters (≥ 5 cells ≥5 UMIs). For each gene, we used a permutation-based approach to estimate empirical *p*-values: reference allele counts were simulated under the null model, and the observed likelihood ratio was compared to the distribution of simulated ratios. We found that 500 permutations were sufficient for stable p-value estimates (tolerance <0.001). *p*-values were adjusted using the FDR [55]. We noted that the simulation-based *p*-value converged around 400 permutations with a tolerance of 0.001 (S8 Fig). Hence, we ran 500 permutations.

### Detecting group-level changes

ASPEN implements two LRTs to assess temporal allelic changes: group-mean (for changes in mean allelic ratios) and group-var (for changes in allelic variance) across discrete groups. Both tests require cells to be assigned to discrete groups, such as by cluster, cell type, or discretized pseudotime. Beta-binomial parameter estimation, dispersion modeling, and shrinkage are performed at both global (all cells) and group-specific levels for each gene.

**Group-mean test:** To assess changes in mean allelic ratio while controlling for dispersion, the null hypothesis assumes all groups share the same mean and shared (‘global’) dispersion:


$${\text{H}}_{\text{0}}:\;\text{AR}\;\sim \;\text{BetaBinomial}\;\left({\text{m}}_{\text{ij}}|{\text{n}}_{\text{ij}},{\;\hat{\mu }}_{\text{i}},{\overset{―}{\theta }}_{\text{SHi}}^{\;\text{glob}}\right)$$

where ${\hat{\mu }}_{\text{i}}$ denotes the shared-group mean and where $\begin{array}{c} {\overset{―}{\theta }}_{\text{SHi}}^{\;\text{glob}} \end{array}$ is the shared dispersion. The alternative hypothesis allows the mean to vary between groups and dispersion is matched to expression based on shrinkage:


$$\begin{array}{c} {\text{H}}_{\text{1}}:\;\text{AR}\sim \sum_{\text{g}=\text{1}}^{\text{L}}\text{BetaBinomial}\left({\text{m}}_{\text{gij}}|{\text{n}}_{\text{gij}},{\;\hat{\mu }}_{\text{gi}},\;{\hat{\theta }}_{\text{WLgi}}^{\;}\right), \end{array}$$

where ${\hat{\mu }}_{\text{gi}}$ is the group-specific mean and $\begin{array}{c} {\hat{\theta }}_{\text{WLgi}}^{\;}\; \end{array}$ the group-specific shrunken dispersion estimate for gene *i*.

**Group-var test:** To assess changes in allelic variance while holding the mean constant, the null hypothesis assumes all groups share the same mean and dispersion:


$$\begin{array}{c} {\text{H}}_{\text{0}}:\;\text{AR}\;\sim \;\text{BetaBinomial}\;\left(\text{m}|{\text{n}}_{\text{ij}},\;\mu^{\text{glob}},\;{\hat{\theta }}_{\text{WLi}}^{\;\text{glob}}\right), \end{array}$$

where $\mu^{\text{glob}}\;$ is the global mean allelic ratio. The alternative hypothesis allows the dispersion parameter to vary between groups:


$$\begin{array}{c} {\text{H}}_{\text{1}}:\;\text{AR}\sim \;\sum_{\text{g}=\text{1}}^{\text{L}}\text{BetaBinomial}\left({\text{m}}_{\text{gij}}|{\text{n}}_{\text{gij}},{\;\mu }^{\text{glob}},\;{\hat{\theta }}_{\text{WLgi}}^{\;}\right) \end{array}$$

where $\begin{array}{c} {\hat{\theta }}_{\text{WLgi}}^{\;}\; \end{array}$ is the group-specific shrunken dispersion estimate.

For both tests, maximum likelihood estimation was used to fit the model parameters, and statistical significance was determined by a likelihood ratio test with *p*-values evaluated from the $\chi_{\text{1}}^{\text{2}}$ distribution (1 degree of freedom). Multiple testing correction is performed using the Benjamini-Hochberg FDR procedure [55].

### Generation of simulated data

To simulate single-cell RNA-seq data, we estimated model parameters using the brain organoid dataset from male Bl6 x Spret hybrids [34]. This dataset included 3,269 cells (clone four), spanning three cell types. The top 2,000 most variable genes identified using getTopHVGs from scater (v1.28.0) [56], and parameters were estimated by fitting a zero-inflated negative binomial model (ZINB-WaVE model, v.1.22.0) [57] to the total count matrix, specifying K = 2 latent cell-level covariates and using default values for other settings.

Reference allele counts were simulated using VGAM (v.1.1-9) [58] by drawing beta-binomial counts from the simulated total count matrix. To assess ASPEN’s ability to detect genes with allelic imbalance, we generated multiple datasets with allelic ratios set to {0.10, 0.30, 0.35, 0.40, 0.42, 0.45, 0.46, 0.47, 0.48, 0.49, 0.50, 0.51, 0.52, 0.53, 0.54, 0.55, 0.57, 0.60, 0.65, 0.70, 0.90}. The dispersion estimates from real data were kept fixed for the respective gene in each simulation. The simulation for each group was repeated five times to improve technical reproducibility.

To simulate temporal or pseudotime scenarios, we again used the ZINB-WaVE total count matrix. Cells were processed using Seurat (v. 4.0.5) [59]. Counts were log-normalized and scaled to 10,000 counts, followed by PCA on all genes. The top 20 principal components were used for Louvain clustering (FindClusters), and UMAP was performed for visualization. Pseudotime was defined by rescaling PC1 coordinates between 0 and 1, then dividing cells into three equal-sized bins (tertiles).

To test detection of temporal changes, beta-binomial counts were simulated for three bins. In the first scenario, the mean allelic ratios were set to {0.45, 0.5, 0.55} with constant dispersion {1 × 10^-3^, 1 × 10^-3^, 1 × 10^-3^} across bins. In the second scenario, mean ratios were held constant (0.5) while dispersion varied across bins {1 × 10^-3^, 0.05, 0.2}. In control simulations, both mean and dispersion were fixed (0.5, 1 × 10^-3^) in all bins.

For assessing the robustness of random monoallelic expression (RME) detection, we sampled total counts from real RME genes and subsampled columns to create datasets with varying cell numbers (n = 10, 20, 50, 100, 250, 500, 1000). Each subsampling was repeated 30 times, with a minimum of 500 genes per set. To mimic RME, allelic counts were sampled using rbetabinom.ab in VGAM (v.1.1-0) [58] with shape parameters (alpha, beta) obtained from ASPEN estimates for real RME genes and limit.prob set to 0.5.

### Detecting allelic imbalance in the simulated data

We applied scDALI (v0.1.0) [24] in homogeneous (Hom) mode to the simulated counts to evaluate the global allelic imbalance. In ASPEN, we used the mean test following the dispersion modeling and shrinking steps. For both methods, we tested the observed allelic ratio against the null hypothesis of AR = 0.5. *P*-values were adjusted for multiple testing via the false discovery rate (FDR). To assess the performance of the tests, we stratified genes by their expression levels based on transcript per million (TPM) values and allelic dispersion. The top 10% of genes fell into the high expression group (log(TPM) > 8), the next 50% to the medium expression group (log(TPM) < 8), and the remaining genes were in the low expression group (log(TPM) < 3). The TPM values were obtained by running calculateTPM from scuttle (v1.10.2) [56] on the total count matrices. Genes were partitioned based on allelic dispersion as follows: genes whose dispersion was less than 0.005 were assigned to the low-dispersion group, and a threshold of 0.45 was selected to assign genes to either the medium- or high-dispersion group. For differential allelic expression benchmarking (*group-mean*), scDALI was used in heterogeneous (Het) mode and allelicRatio function was used from Airpart [23] with DAItest set to TRUE.

### Bulk RNA-Seq data processing

Mouse fibroblast RNA-Seq fastq files to evaluate read mapping strategies were downloaded from GSE193728. To compare the distribution of genes with low dispersion between single-cell and bulk RNA-seq data, CD8 + T-cell data from male Bl6 × Spret hybrid data were obtained from GSE126770. The reads were trimmed via Trimmomatic [60] in paired-end mode and mapped to the combined genome index via STAR (v2.7.6a) [50]. To achieve full-length read alignment, the insertions, soft-end clipping and splicing modes were turned off (--outBAMcompression 6 --outFilterMultimapNmax 1 --outFilterMatchNmin 30 --alignIntronMax 1 --alignEndsType EndToEnd --outSAMattributes NH HI NM MD AS nM --scoreDelOpen -1000 --scoreInsOpen -1000). Reads mismatched (flagged as ‘NM’) and reads overlapping diverse strain-specific regions that were enriched for recently transposed long interspersed nuclear elements (LINEs) and long-terminal repeat (LTR) elements [61] were removed. Reads were counted via Rsubread (v2.4.3104) [62] with the following parameters: isPairedEnd = T, allowMultiOverlap = T, and countMultiMappingReads = F. To normalize for sequencing depth, size factors were estimated via total counts (sum of allelic counts) with DESeq2 (v1.30.1) [30] and used to adjust allelic counts.

### scRNA data preprocessing

CD8 + T-cell data for naïve, day 7 and day 40 postinfection samples were obtained from the GSE164978 dataset (SRA files SRR13450127 and SRR13450128). Fastq files for mouse brain organoids generated from female Bl6 × Cast and male Bl6 × Spret F1 hybrids were downloaded from GSE268332. Files were mapped to the respective strain genomes via STARSolo (v.2.7.6a) [63] (--soloType CB_UMI_Simple --soloUMIlen 12 --soloCBstart 1 --soloCBlen 16 --soloUMIstart 17 --soloCBwhitelist <(zcat ${dir}/align/3 M3M-february-2018.txt.gz) --soloFeatures Gene --outSAMattributes NH HI nM AS CR UR CB UB GX GN sS sQ sM NM MD --outBAMcompression 6 --outFilterMultimapNmax 1 --outFilterMatchNmin 30 --alignIntronMin 20 --alignIntronMax 20000 --alignEndsType EndToEnd --outFilterMismatchNoverReadLmax 0.001 --scoreDelOpen -1000 --scoreInsOpen -1000). To evaluate Expectation Maximization strategy for the multimapping reads allocation, we added the following flags in STARSolo: --outFilterMultimapNmax 3 and --soloMultiMappers EM. The total count matrix was produced by combining maternal and paternal allelic counts. Genes that were expressed in fewer than 10 cells were removed from further analysis.

### Single cell data allelic testing in CD8 + T cells

We conducted ASPEN allelic imbalance and variance tests on data from F1 mouse brain organoids and CD8 T cells. We examined 5,095 genes across 723 (naïve), 400 (day 7), and 582 (day 40) cells. The total count matrices for each time point were merged. Size factors were calculated using computeSumFactors from scran (v.1.28.2) [64] by pooling similar groups of cells together (we used the original time points for cluster assignment). The same scaling was applied to both the total and reference allele counts. Initial beta-binomial parameters were estimated, and dispersion modeling and shrinkage were conducted with raw counts. The expected allelic ratio was adjusted to the empirical ratio of 0.52 for the allelic imbalance test to reflect an alignment bias toward the reference genome (S9A Fig). Testing was then performed on the normalized counts, selecting genes that had at least 5 cells with at least 5 mapped reads. We ordered the datasets by time points (naïve, day 7, and day 40 post-infection) to assess dynamic changes in the allelic ratio distribution and used this as a discrete temporal assignment. P values for the ASPEN tests were FDR-adjusted.

### Pseudobulked allelic testing in CD8 + T cells

Merged CD8 + T-cell counts were processed in Seurat (v. 4.0.5) [59]. We performed principal component analysis (PCA) on log-normalized data and scaled it to 10000 counts using all genes. The top 20 principal components (PCs) were selected for downstream Louvain clustering as implemented in the FindClusters function (resolution = 0.9), and low-dimensional representations were obtained through uniform manifold approximation and projection (UMAP). This process produced three clusters per time point, which were then used to aggregate the total counts as pseudoreplicates within each cell state (in lieu of the biological replicates) (S9B Fig). The same cell assignment was used to pool the reference allele counts. Size factors were estimated via total counts with DESeq2 (v1.30.1) [30] and were applied to scale the total and reference allele counts in parallel. Genes with a minimum of 10 reads across all clusters within the sample were retained for allelic imbalance testing. We checked for mapping bias in the pseudobulk dataset, revealing a slight skew toward the Bl6 allele, which was accounted for by adjusting the expected ratio (S9C Fig). P-values for the allelic imbalance test obtained from ASPEN were FDR-adjusted.

### Analysis of mouse brain organoid single-cell RNA-seq data

To obtain initial beta-binomial parameters and shrunk dispersion values from CD8 T cells, we used the same procedure as for the male Bl6 × Spret hybrid data. For allelic imbalance testing, we adjusted the allelic ratio value under the null hypothesis to 0.54. Using beta-binomial shape parameters, we defined genes with α < 1 OR β < 1 as monoallelically expressed.

### Allelic imbalance in pseudobulked counts from mouse brain organoids

Single-cell Bl6 alleles and total counts from four female Bl6 × Cast F1 hybrids were aggregated by clonal origin to generate pseudobulk replicates. We estimated size factors using total counts with DESeq2 (v1.30.1) [30] and applied them to scale the total and Bl6 allele counts. Genes with fewer than 10 total counts across the pseudoreplicates were excluded from further analysis. Differentially expressed genes between RGC and IPC cell types were identified based on the total pseudobulked counts using nbinomWaldTest function from DESeq2 (v1.30.1) [30].

### Identification of random monoallelic expression

We identify RME expression patterns using the shape parameters (α and β) of the beta-binomial distribution. When either α or β falls below one, the distribution becomes skewed towards one allele. The hallmark of RME emerges when both α and β are less than one and are approximately equal, producing a U-shaped allelic density – indicating that individual cells express either the maternal or paternal allele but rarely both. We identified incomplete X chromosome inactivation in genes that exhibited biallelic expression based on the following criteria: mean allelic ratio between 0.25 and 0.75 in at least 15% of cells, with a minimum of five cells.

### Allele-specific TF binding motif enrichment analyses

Processed open chromatin data for T cells at day 7 post-LCMV infection were obtained from GSE164978 [32]. scATAC peaks overlapping the promoter regions (+/-1 Kb) around TSS of the allelically imbalanced genes (ASPEN-mean FDR < 0.05 & |log2FC| ≥ 1) were selected. Coordinates of the corresponding regions on the Spret allele were obtained by lifting over the Bl6 promoter region coordinates using the chain file generated above and USCS liftOver [49]. To calculate the motif enrichment scores, FIMO (v.5.5.4) [65] was run for Bl6 and Spret sequences of their respective promoter regions. The sequences were extracted from either the Bl6 or Spret genomes using the getfasta command from bedtools [66]. Selecting motif with significant enrichment on both alleles (*p* <$\text{1}\times {\text{1}\text{0}}^{-\text{4}}$), the differences between the FIMO log odds scores were calculated.

### Gene stability analyses

A list of genes ranked by the stability index was obtained from Lin et al [33]. Genes with Stability Index ≥ 0.8 were defined as Stably Expressed Genes (SEGs) and genes with Stability Index < 0.8 – as non-SEGs. We matched the SEGs and non-SEGs by their expression level and the number of available cells by performing the nearest neighbour search using MatchIt (v.4.5.5) [67] with parameters set to method = “nearest” and distance = “glm”. Housekeeping genes were obtained from HRT Atlas v1.0 [36] database by selecting either “Embryonic Stem Cells” or “Spleen bulk tissue”. Essential and non-essential genes for T-cell clonal expansion were selected in the same manner. Matching was performed based on the gene expression alone.

### Functional enrichment analysis

Gene Ontology (GO) term enrichment analysis was performed in clusterProfiler (v4.8.3) [68] via the org.Mm.e.g.db database (3.17.0) [69]. We used the enrichGO function to identify enriched biological processes in genes with low dispersion (${\hat{\theta }}_{\text{i}}\;<\;\text{0}.\text{001}$) in the T-cell data collected on day 7 post-LCMV infection. All genes with sufficient coverage from the same time point were used as the background for GO enrichment.

### Availability of data and materials

ASPEN is available as an R package at https://github.com/ewonglab/ASPEN

The source code and data used to produce the results and analyses presented in this manuscript are available from Github repository: https://github.com/ewonglab/ASPEN_manuscript. Single-cell RNA-seq data for CD8 T cells from naïve, day 7, and day 40 LCMV Armstrong infection samples were downloaded from the GSE164978 dataset (SRA: SRR13450127, SRR13450128) [32]. Bulk RNA-Seq data for CD8 + T cells 7 days after LCMV Armstrong infection were downloaded from GSE126770 [31]. Fastq files for bulk RNA-seq of mouse fibroblasts from four F1 hybrids (Bl6 × Cast, Bl6 × Molf, Bl6 × Pwk, and Bl6 × Spret) were obtained from GSE193728 [70]. Single-cell RNA-seq data for mouse brain organoids (Bl6 x Cast and Bl6 x Spret) were downloaded from GSE268332 [34]. Variant call files (release 5) of the four wild-derived mouse strains were obtained from the Mouse Genome Project [47]. A list of recently transposed long interspersed nuclear elements (LINEs) and long-terminal repeat (LTR) elements in the strain-specific genomes was downloaded from the original publication [61].

## Supporting information

S1 Fig Comparing two F1 hybrid reads mapping strategies: SNPs level variation and SNPs and indels level variation between the parental genomes. Schematic for F1 breeding strategy (top panel). The difference in the number of reads mapped to the combined genomes generated with either SNPs only or both SNPs and indels using fibroblast cells from four mouse F1 hybrids – Bl6 × Cast, Bl6 × Molf, Bl6 × Pwk and Bl6 ×* *Spret (bottom panel).
(TIF)

S2 Fig Genes with low dispersion involved in broad cellular functions. (A) GO term enrichment analysis using the genes with low dispersion. Enriched gene ontology biological process function categories for genes with low allelic dispersion in T-cells day 7 post LCMV infection scRNA dataset (B) Mean expression and proportion of non-zero cells between the stably expressed genes (SEGs), identified in Bl6 × Cast F1 brain organoids dataset, and background, genes matched by expression level and number of non-zero cells (two-sided Wilcoxon rank-sum test) (complementary to Fig 2C).
(TIF)

S3 FigDetecting genes with allelic imbalance using ASPEN.(A) Performance comparison between ASPEN and scDALI using simulated data (complementary to Fig 3C). Line plots showing TPRs calculated at FDR = 0.001 for genes stratified by gene expression and allelic dispersion levels (low dispersion – θ < 0.005, medium – θ ≥ 0.005 & θ < 0.4 and high – θ ≥ 0.4) across groups with varying levels of deviation from the mean AR = 0.5. Only datasets simulated with a mean AR of 0.5 ± 0.2 are shown. (B) ASPEN runtime (top) and memory usage (bottom) for different numbers of cells and genes (calculations were restricted to one core using a 6 CPU Intel Xeon, 64GB RAM machine). (C) Correlation of -log_10_FDR values from ASPEN mean test ran on the single-cell counts (x-axis) and pseudobulked counts (y-axis) (complementary to Fig 3E). Analyses were performed in mouse brain organoid cells from female Bl6 × Cast F1 hybrids (left panel): radial glial cells (RGCs, n = 1,072) and intermediate progenitors (IPCs, n = 853); and in CD8^+^ T cells from male Bl6 × Spret F1 hybrids (right panel): the naïve state (n = 221) and day 7 (n = 525) after LCMV infection. (D) Relationship between allelic ratios dispersion and mean gene expression in organoid cells from female Bl6 × Cast F1 hybrid (left panel) and CD8 T-cells from male Bl6 × Spret F1 hybrid (right panel). The dashed line separates genes with low variation which are excluded from the shrinkage procedure. The local regression model fit is shown in light blue. (E) Top 50 genes with significant allelic imbalance (ASPEN mean FDR < 0.05) identified in T-cells from male Bl6 × Spret F1 hybrids and consistently expressed in all three cell states. (F) Correlation between allelic ratio and changes in motif score for the variants inside the promoter peaks (+/- 1Kb from TSS). Allelically imbalanced genes with strong bias to either Bl6 or Spret allele are selected (ASPEN-mean FDR < 0.05, |log2FC| ≥ 1).(TIF)

S4 Fig ASPEN workflow. scRNA data pre-processing steps, including the generation of the F1 index file for ASE reads mapping (top row) and ASPEN pipeline (bottom row). Tests evaluating allelic variation (deviation from the expected level of dispersion for genes with similar expression – bb_var, allelic variance changes across the groups – group_var) are performed using raw counts. For the group-level tests (group_mean and group_var), dispersion stabilization steps are to be repeated for each group and across all cells. The counts normalization is performed outside of ASPEN. We used computeSumFactors from scran (Lun., et al. 2016). The minimum coverage threshold for the genes is set to a minimum of five reads in at least five cells (min_counts = 5, min_cells = 5). We find this to be sufficient to reliably identify ASE patterns in most datasets, however for the differential changes in variance we recommend increasing the minimum number of cells to 15 (min_cells = 15).
(TIF)

S5 Fig Detecting group-level changes in allelic patterns during early neurogenesis. (A) Mean expression and proportion of non-zero cells between the housekeeping genes (HKGs), identified in Bl6 × Cast F1 brain organoids dataset, and background, genes matched by expression level and number of non-zero cells (two-sided Wilcoxon rank-sum test) (complementary to Fig 4D). (B) Allelic ratio distribution in three simulated scenarios: changes in mean AR (with dispersion parameters held constant), changes in AR variance (with mean parameters held constant), and a control group (where both mean and dispersion parameters are constant). The reference allele counts were simulated by drawing from a beta-binomial distribution parameterized by the bin-wise total counts, mean (μ), and dispersion (θ) for the respective bin as indicated. (C) Comparing the accuracy in detecting allelic ratio change across the groups between ASPEN, scDALI and Airpart in different gene expression groups. (D) Comparing the accuracy in detecting changes in allelic variation in different gene expression groups. (E) Global overview of the genes with group-level allelic variation in differentiation from radial glial cells (RGCs) to intermediate progenitor cells (IPCs) in female Bl6 x Cast F1 hybrids. Genes are partitioned by changes in variance (n = 158) and both mean and variance (n = 148). (F) Genes with differential allelic patterns between RGCs and IPCs are associated with either Intellectual Disability/Autism Spectrum Disorders (ID/ASD, SFARI score 1) or with neuroanatomical phenotypes in mice.
(TIF)

S6 Fig Evaluating monoallelic ASE patterns in mouse brain organoids from female Bl6 x Cast hybrid. (A) Examples of AR distribution in genes with monoallelic expression (defined using beta-binomial shape parameters α < 1 or β < 1). Genes showing consistent monoallelic expression on either of the alleles include imprinted and autosomal genes. FDR values indicate deviation from balanced ASE (ASPEN mean test). (B) Allelic ratio distribution of genes with incomplete X chromosome inactivation in female Bl6 × Cast F1 hybrid. (C) Allelic ratio distribution plots for the autosomal genes with random monoallelic expression (RME) found in the brain organoid data (complementary to Fig 5D). (D) True positive rate for RME detection in the simulated data. 500 genes were simulated with different numbers of cells (n = 10, 20, 50, 100, 250, 500, 1000) and stratified by gene expression. (E) Median expression and median number of non-zero cells between RME and non-RME genes. Median expression: RME – 0.96, non-RME – 0.97, D = 0.182, p = 0.08, two-sided K-S test. Median number of cells: RME - 526, non RME – 654, D = 0.279, p = 0.001, two-sided K-S test. (F) List of autosomal genes identified as RME (defined using beta-binomial shape parameters α < 1 and β < 1 and |α – β| < 0.5) with evidence of previous reports in the literature.
(TIF)

S7 Fig Allelic variation patterns during T-cell activation. (A) Gene Ontology (GO) term enrichment analysis result for genes with allelic variance different from the expected for similar expression levels (ASPEN var: FDR < 0.05). The analysis included genes across all three states (naïve, day 7 and day 40). The genes were divided into lowly dispersed (empirical dispersion is less than common) and highly dispersed (empirical dispersion is greater than common) groups. (B) Mean expression and proportion of non-zero cells between the housekeeping genes, identified in lowly dispersed T cells, and background, genes matched by expression level and number of non-zero cells (two-sided Wilcoxon rank-sum test) (complementary to Fig 6B). (C) Abundance of genes essential for the T-cells expansion (marked in red) in day 7 post LCMV infection scRNA dataset. The boxplot shows mean dispersion estimation between essential genes and background, a set of genes matched by gene expression (two-sided Wilcoxon rank-sum test). (D) Mean allelic ratio across the cell states for genes with significant deviation from allelic trend (complementary for Fig 6B) (E) Boxplots showing the mean ASE (two-sided Wilcoxon rank-sum test, ‘****’ *p* ≤ 1 × 10^-4^, ‘**’ *p* ≤ 1 × 10^-2^) for genes with varying temporal distributions: *Naca* (changes in allelic variance), *Actb* (changes in allelic mean) and *Trbc1* (changes in both allelic variance and mean) (complementary to Fig 6E).
(TIF)

S8 Fig Selecting appropriate number of permutations with tolerance < 0.01. We used data simulated with mean AR = 0.42 and tested different number of permutations (100 for each step) to calculate the *p*-value difference for one point. A tolerance below 0.01 was reached after 400 permutations.
(TIF)

S9 Fig Allelic imbalance analyses using single-cell and pseudobulked counts in mouse T-cells from male Bl6 x Spret hybrid. (A) Global AR distribution across all genes in the T cells dataset (scRNA counts), showing a slight bias at 0.52 towards the BL6 allele. (B) UMAP showing clustering of the T cells within each cell state that was used for the pseudo-bulk aggregation (C) Global AR distribution across all genes in the mouse T-cells dataset (male Bl6 x Spret hybrid, using pseudo-bulked scRNA counts), showing a slight bias towards the BL6 allele.
(TIF)

S10 Fig Expectation Maximization (EM) strategy for the multimapping reads inflates the number of genes with low expression. (A) Inclusion of multi-mappers leads to ~2-fold increase in the number of allelically resolved reads. Using the T-cell data, the reads were mapped against the Bl6 × Spret F1 genome. The average increase in the number of mapped reads was from 34.3% (unique) to 60.6% (unique + multiple). (B) Global AR distribution in genes quantified using multimapping and unique reads (top row) and unique reads only (bottom row). Using multi-mappers for gene quantification leads to higher dispersion around the mean AR. (C) ASPEN-mean test results for two gene categories: including the multimapping reads and using the unique reads only. The total number of genes (after quantification), the number of genes after applying the minimum coverage threshold (at least five cells with a minimum of five reads), the number of genes with allelic imbalance (ASPEN-mean FDR < 0.05) and the overlap between the two groups are shown. A smaller fraction of allelically imbalanced genes in the first gene category suggests that using multimapping reads does not necessarily lead to better accuracy in detecting allelic imbalance.
(TIF)

S1 Appendix Supplemental Results. Result of including multiple mapping reads.
(DOCX)

S2 Appendix Supplemental Tables. Supplemental tables contents and descriptions (Tab 1). The breakdown of genes tested based on the original and shrunk dispersion estimates shown in Fig 3B (Tab 2). Imprinted and sex chromosome genes in male mouse brain organoid Bl6 x Spret hybrid (Tab 3). Results of the allelic imbalance and variance tests using single-cell counts from male Bl6xSpret hybrid (Tab 4 - CD8 Naïve T-cells, Tab 5 - CD8 T-cells day 7 post infection, Tab 6 - CD8 T-cells day 40 post infection). Perfromance comparison between ASPEN group-mean, scDALI-Het and Airpart DAI tests shown in Fig 4G and Supp. Fig 5C (Tab 7). Perfromance metrics for ASPEN group-var test shown in Fig 4H and Supp. Fig 5D (Tab 8). Results of the tests for the differential changes in the allelic ratio mean and variance using single-cell counts from female mouse brain organoid Bl6xCast hybrid (Tab 9). Results of the differential gene expression analyses between RGCs and IPCs in brain organoids using pseudo-bulked counts (Tab 10). Genes with monoallelic expression in female brain organoid Bl6 x Cast hybrid (Cortical neurons - Tab 11, IPCs – Tab 12, RGCs – Tab 13). Results of the tests for the differential changes in the allelic ratio mean and variance using single-cell counts from male Bl6 x Spret hybrid (Tab 14 – t-cells). Results of the allelic imbalance test using pseudobulked counts from male Bl6 x Spret hybrid (Tab 15 - CD8 Naïve T-cells, Tab 16 - CD8 T-cells day 7 post infection, Tab 17 - CD8 T-cells day 40 post infection). Results of the allelic imbalance test using pseudobulked counts from female Bl6 x Cast hybrid (Tab 18 – cortical neurons, Tab 19 – IPCs, Tab 20 – RGCs).
(XLSX)
