# Supplementary material for: ASPEN: Robust detection of allelic dynamics in single cell RNA-seq
Source: PLoS Comput Biol. 2025 Dec 19;21(12):e1013837. doi: 10.1371/journal.pcbi.1013837 (PMC12774380; doi:10.1371/journal.pcbi.1013837)
Supplement: S1 Appendix — Result of including multiple mapping reads. (DOCX) [file pcbi.1013837.s011.docx]

**Supplemental Results**

**Result of including multiple mapping reads**

We tested the inclusion of weighted multimapping reads using STARSolo’s EM method to probabilistically assign multimappers based on gene expressed across all cells (Kaminow, 2021)(**Methods**). Inclusion of multimapping reads resulted in recovery of ~2-fold of allelic reads, from 26.7M to 47.2M on average, based on the B6xSpret F1 data (**S10A Fig.**). However, most of those reads do not overlap an informative variant. We detected increased dispersion (**S10B Fig.**). Despite evaluating a greater number of genes when using both the multimapping and unique genes, 27.7% fewer genes had significant allelic imbalance (ASPEN-mean FDR < 0.05, n=1,167) than those identified using unique counts (**S10C Fig.**). Our observations suggested that the inclusion of multi-mapping reads leads to lower sensitivity in allelic imbalance detection.
